# The Types of Teeth in Disease

**Published:** 1887-07

**Authors:** James F. Rymer


AMERICAN JOURNAL

—OF—

DENTAL SCIENCE.

VOL. XXI. THIRD SERIES—JULY 1887. NO. 3.

ARTICLE I.

### THE TYPES OF TEETH IN DISEASE.

BY JAMES F. RYMER, M. R. C. S.

[A paper read before the Students' Society of the National Dental College.]

There are three reasons why I thought this subject would be worth bringing forward:

Firstly, Our text-books and journals do not contain a large amount of information, and what there is is scattered over a period of 40 years; and that by collecting these fragments together they might constitute a homogeneous whole bearing upon the question.

Secondly, As undoubtedly the teeth are affected by many diseases, it appeared to me that if we took the trouble to examine more thoroughly the forms and defects of the teeth, it might in some ill-marked disease prove most useful, by giving to the diagnostician another valuable symptom.

Thirdly, As many of our younger members have not yet attended the Medical and Surgical Wards at a General

Hospital, and so have not had opportunities of studying the commoner diseases, such as gout, syphilis, &c., I thought by knowing what kind of teeth to expect in those everyday complaints, and which are seen in many of the patients who come to this Hospital, they would become more thoroughly acquainted with their peculiarities, and so, to a great extent, be able to spot gout, syphilis, rickets, struma, &c. I do not intend to (nor could I if I wished) enter at all deeply into the subject; but in the endeavour to point out some special features of the teeth, which we ought all to be able to recognise, I have been obliged to put in two or three queries, hoping that some of our more experienced and learned members will be able to fill them in for me. With these remarks, I commence with

GOUTY TEETH.

Before describing these teeth, I will hand round a model of a well-marked gouty case. This patient had tophi of the ears, reedy nails, and a good gouty history. It may be interesting to mention that Dr. Graves first called attention to the ground down condition of the teeth as long back as 1836 in the *Dublin Medical Journal*, and there explained that the grinding is due to an irritable condition of the dental nerves. All of us, I think, will agree that this does not explain the cause. Again, Dr. Horatio Donkin, in the *British Medical Journal* for February 21st, 1880, points out the following case of a family:—The father suffered much with gout; the mother's ancestors were full of gout. This couple had eight children; all of them had their teeth much worn down, and, Dr. Donkin remarks, when asleep the grinding produced an impressive non-harmonious concert. In the Norse type the teeth are, as a rule, solid, blunt, and thick at the edge, due to the wearing down; dark in centre, due in many cases to a formation of dirty secondary dentine; the incisors are more worn than the canines or molars. Gouty teeth have a tendency to work out without caries, from an osteitis extending from the neck along and towards the end of the root. The gums are often retracted, and the mouth offensive, the latter being due to gouty dyspepsia. According to Dr. Milner Fothergill, there is often a well-marked osteal growth along the fang of the canine. A question now arises, What is the cause of this wearing away of tooth substance? Is it some malformation in the enamel and dentine? Or is it due to simple attrition, the result of some reflex irritation, as dyspepsia, &c.? It seems most probable that it is a combination of the two, for many people grind their teeth (as children with worms, &c.), yet their teeth do not wear down to the extent found in gouty subjects. Out of a large number of "gouty mouths" that I have examined at "Guy's," without exaggeration 80 per cent. either had gout or come from a gouty family.

Before leaving the subject of gouty teeth, I would like to mention rather a novel, at the same time an interesting case. A female patient, aged 30, came to the hospital one Friday, about a month ago, complaining of intense pain (not of a shooting character) in the right upper lateral tooth. Her teeth were distinctly gouty; I examined the tooth carefully, as did also the dental surgeon for the day, but we failed to find any caries or crack. It then occurred to me that it was a manifestation of gout confined to this tooth. I applied (by direction) a strong counter-irritant, chloride of zinc, around the gum and upon the tooth. I saw her a week after, when she said that all pain had disappeared in 24 hours after the application. I may add that the laterals were worn away considerably less than the centrals, and certainly there was no sign of sensitive dentine or pulp exposure. Was this a case of gout? If not, what was it?

### STRUMOUS TYPE OF TEETH.

Scrofulous or strumous people often present signs of imperfect nutrition and development, their teeth being large, and of chalky appearance, and often have milk-white spots on the surface, and mostly upon the buccal surface. These indicate defective development of the enamel, with less gelatine than normal, and so the enamel is more easily acted upon by exposure to acid secretions, or to other external irritants. As these patients frequently suffer from "strumous dyspepsia," it is in those faulty spots that caries usually commences. The patient from which I obtained this model was of very strumous appearance, and although the teeth are not large, the model shows a typical case of strumous caries. The jaws are generally large in proportion to the teeth, and so crowding and irregularity is avoided.

### PHTHISICAL TYPE OF TEETH.

Here the teeth are small and irregular, the palate is long, the central incisors lean towards each other, producing the "rabbit jaw;" or the teeth may be set at an obtuse angle, forming the "horse jaw." The teeth are very prone to caries, most commonly the upper incisors, especially the laterals (by some this is considered the test tooth for hereditary phthisis.) Decay begins at puberty and at the sides; and so differs from strumous teeth both in size and site of decay. Corfe says that decay is due to the imperfect flow of blood in the vessels of the teeth (!).

### RICKETY TEETH.

Although there is nothing peculiar in the type of these teeth, yet there is something quite characteristic which I have not yet seen mentioned in our medical or dental books. It is as follows:—The temporary teeth are erupted in the normal manner, but at about 2½ to 3 years of age they loosen without any visible inflammation of the gums and drop clean out, the roots presenting no signs of any absorption. Through the kindness of Mr. Charters Symonds, of "Guy's," I had the opportunity of examining quite twenty-four cases of children suffering from rickets. It was quite an exception to find all the teeth present; in some cases both the upper and lower incisors had loosened and dropped out. This rough model was taken from a squealing baby, æt. 2½.

The mother came to the hospital and asked me the reason of the teeth coming out. I examined the patient's tibiæ and found well-marked curves, the lower ends of the radi and ulnæ were enlarged, the anterior fontanelle was also large; and naturally I told the mother that rickets was the cause. She then told me that a year previously the child had been treated at the Middlesex Hospital for rickets. The permanent teeth are often, erupted later than usual and the centrals frequently have two small notches showing lines of development. They are either carious when erupted, or else decay shortly afterwards; hence it is important to recognise rickets by the temporary teeth, for then the parents can be told what condition the permanent will very probably be in. Rickets is most common in the extreme poor or in the later members of large families.

### STOMATITIC TYPE.

Under this head one has to jumble together divers diseases, such as scarlet fever, measles, convulsions, jaundice, &c., which all seem to produce the same defects in the teeth. The temporary teeth, although unaffected, are often erupted later than usual; but greater interest lies in the permanent teeth, for during the period of the illness there is an arrest in the development of the enamel, and the dentine is seen to be a dirty brown colour. Thus, if a child is ill during the first year, and then recovers, the tip and half the lower surface of the central incisor teeth is devoid of enamel; and if at a year and a-half, there will be a ring of exposed dentine near the cervical margin.

MERCURIAL TEETH

are closely allied to the above. Many people are dubious whether it is possible to distinguish a mercurial tooth from one due to stomatitis, but there seems to be one or two points characteristic; generally it is the lower portion of the enamel that is defective, differing from the stomatitic tooth, which may be faultly at any portion of the crown. The surface is seamed and jagged, yellow and irregular in form; the dentine frequently has rings of enamel on its surface, giving it a pitted appearance. In order to distinguish between a congenital and an acquired mercurial tooth, the tip or cutting edge is a sure guide (before the age of 20), for in the former the cutting edge is always devoid of enamel, whilst in the latter the enamel in this situation is often perfect for a distance of one or two lines.

As to the cause. Is this condition due to a simple stomatitis? Or is the pitting, &c., due to the removal of the forming enamel by the absorbing power of the mercury? Certainly many infants have convulsions entirely apart from dental irritation or the popular term "cutting the teeth," for convulsions in infants are due to a variety of causes, as diarrhœa, colds, &c., but still ignorant mothers blame the poor teeth and administer *ad lib.* "Stedman's Teething Powders" and other quack remedies, with the result that the previously healthy developing enamel is in some way affected by the mercury, either by absorption or by setting up a stomatitis. The six-year-old molars are the test teeth for mercury.

SYPHILITIC TEETH, or commonly known as "Hutchinson's Teeth," are now to be mentioned. Although by far the most important, they are the most easy to recognise, and are tolerably familiar to us all; therefore only a few words about them will suffice. A very interesting discussion about them will be found in

*The Lancet*, vol. i., 1876, which took place before a dental society, where the discoverer, Mr. Jonathan Hutchinson boldly defended his pet teeth, in opposition to the opinion expressed by members of the dental profession, who contended that this peculiar form of tooth was independent of syphilis, and when people were known to have been born with the disease, it was a mere fluke if their teeth were altered in form. Happily, now the discoverer has convinced all that syphilitic teeth are always a reliable guide. The chief points are: notching of the two centrals, which are always symmetrical; if the notching is not confined to the centrals, then the cause is most likely to be mercury, &c. The teeth are small, of dirty grey colour, and narrower at the cutting edge than at the neck. Again, on some of the teeth, other than the upper centrals, are to be seen several grooves along the surface, giving rise to the so-called "serrated teeth;" or there is a central projection, "pegtop teeth." The grooves and other characteristic peculiarities begin to get worn down by the age of 20; hence they are far more difficult to diagnose with certainty after that age. The temporary teeth are never affected, but are normal. Why? For the specific inflammation does *not* take place in utero, but calcification does.

There is yet one more rare form of tooth. I allude to "Mercurio-Syphilitic." It is only a cross between the mercurial and syphilitic types; by knowing these, it is easy to recognize. I had the luck to see a good example of this type of tooth at this hospital about a year ago.

I have just recently had the opportunity of examining a somewhat unique mouth, exhibiting very clearly three distinct forms of teeth—viz., syphilitic, mercurial and rachetic. The patient, a girl æt. 10, was brought to the National Dental Hospital from the "Home for Waifs and Strays." Unfortunately, as no person appeared to claim the child, no history could possibly be obtained.

The patient had only one upper permanent incisor which was peg-shaped, but had a rough irregular cutting edge; the lower incisors were present, but so malformed as to appear as simple rough brown projections through the gums. I may add that the child had a large square head and face, the latter being of a decidedly "earthy" appearance; the elbow joints were much enlarged, evidently due to some early diseased action, and the child was very short in stature. These and other conditions pointed to congenital syphilis.

Now, in favour of mercury: The four six-year-old molars presented the typical mercurial appearance which I have elsewhere spoken about. In favour of rickets: All the deciduous set of teeth had disappeared, leaving the gums smooth and healthy, as pointed out in describing rickety teeth. In addition to this the upper jaw was small, due to arrested development; the lower jaw, although appearing larger, was of normal size. This disproportion between the size of the jaws has also a rickety significance.

Lastly I may add that the recently erupted permanent bicuspids were normal in number and of healthy appearance.—*London Dental Record.*

